# Schistosomiasis Among Female Migrants in Non-endemic Countries: Neglected Among the Neglected? A Pilot Study

**DOI:** 10.3389/fpubh.2022.778110

**Published:** 2022-03-09

**Authors:** Sílvia Roure, Olga Pérez-Quílez, Xavier Vallès, Lluís Valerio, Israel López-Muñoz, Laura Soldevila, Ariadna Torrella, Gema Fernández-Rivas, Anna Chamorro, Bonaventura Clotet

**Affiliations:** ^1^North Metropolitan International Health Unit Programa de Salud Internacional del Institut Català de la Salut (PROSICS), Badalona, Spain; ^2^Infectious Diseases Department, Germans Trias i Pujol University Hospital, Universitat Autònoma de Barcelona, Badalona, Spain; ^3^Fundació Institut per la Recerca en Ciències de la Salut Germans Trias i Pujol, Badalona, Spain; ^4^Microbiology Department, Germans Trias i Pujol University Hospital, Badalona, Spain; ^5^AIDS Research Institute-IrsiCaixa Foundation, Badalona, Spain; ^6^Germans Trias i Pujol University Hospital, Universitat Autònoma de Barcelona, Badalona, Spain; ^7^Fundació Lluita contra la Sida i les Malaties Infeccioses, Germans Trias i Pujol University Hospital, Badalona, Spain

**Keywords:** female genital schistosomiasis, imported schistosomiasis, migrants females, neglected, migrants population in Europe, schistosomiasis in non-endemic countries

## Abstract

**Background:**

Schistosomiasis among migrant populations in Europe is an underdiagnosed infection, yet delayed treatment may have serious long-term consequences. In this study we aimed to characterize the clinical manifestations of Schistosoma infection among migrant women, and the degree of underdiagnosis.

**Methods:**

We carried out a prospective cross-sectional study among a migrant population living in the North Metropolitan Barcelona area and coming from schistosomiasis-endemic countries. We obtained clinical, laboratory and socio-demographic data from electronic clinical records, as well as information about years of residence and previous attendance at health services. Blood sample was obtained and schistosomiasis exposure was assessed using a specific ELISA serological test.

**Results:**

Four hundred and five patients from schistosomiasis-endemic regions were screened, of whom 51 (12.6%) were female. Seropositivity prevalence was 54.8%, but considering women alone we found a prevalence of 58.8% (30 out of 51). The median age of the 51 women was 41.0 years [IQR (35–48)] and the median period of residence in the European Union was 13 years [IQR (10–16)]. *Schistosoma*-positive women (*N* = 30) showed a higher prevalence of gynecological signs and symptoms compared to the seronegative women (96.4 vs. 66.6%, *p* = 0.005). Among seropositive women, the median number of visits to Sexual and Reproductive Health unit prior to diagnosis of schistosomiasis was 41 [IQR (18–65)].

**Conclusion:**

The high prevalence of signs and symptoms among seropositive women and number of previous visits suggest a high rate of underdiagnosis and/or delayed diagnosis of *Schistosoma* infection, particularly female genital schistosomiasis, among migrant females.

## Introduction

Schistosomiasis is a parasitic infection which is included in the World Health Organization's list of neglected tropical diseases. In recent decades, migration to Europe from regions where schistosomiasis is endemic has increased the risk of imported infections, which may remain largely underdiagnosed. A recent review study estimates the prevalence of infection in sub-Saharan immigrants living in non-endemic countries at 24% (1). Applying this figure to the estimated number of immigrants coming from schistosomiasis-endemic countries now living in our region (Catalonia), there could be as many as 17,000 infected individuals, most of them undiagnosed. More specifically, in the catchment area of our public health unit (North Metropolitan Barcelona), which serves ~1,300,000 inhabitants (of whom roughly 15% are immigrants from non-EU countries), there could be over 5,000 infected individuals (1, 2). Estimation of the real burden of schistosomiasis is hampered by the lack of an adequate gold standard for diagnosis. Detection of parasite eggs in urine or stool samples has very low sensitivity and serological testing is considered the most cost-effective diagnostic tool in non-endemic countries (3–5).

Schistosomiasis infection may lead to long-term and chronic complications related to the respective organ-specific targets of the two *Schistosoma* species most common in Africa, *S. haematobium* (urogenital tract) and *S. mansoni* (gastrointestinal system). The most characteristic chronic complications include chronic renal insufficiency and bladder cancer, as well as a large number of specific and unspecific morbidities (6). More particularly, infection may produce pathological alterations in the genitalia of women, defined as female genital schistosomiasis (FGS). FGS is mostly caused by *S. haematobium*. However, *S. japonicum* and *S. mansoni* also occasionally affect the genital tract (6). Currently, FGS is considered a common but neglected infection, with estimations of global prevalence ranging from 33 to 75% among infected women. FGS thus potentially affects up to 28 to 64 million among the estimated 85 million of women infected by *S. haematobium* worldwide (7). FGS prevention and treatment is an important component of the sexual and reproductive health care of exposed women. This is particularly concerning since *Schistosoma* infection is easily treatable with an effective, safe and low-cost drug (praziquantel).

This pilot study has two main goals: first, to characterize the clinical manifestations of presumptive *Schistosoma* infection among migrant women, and second, to test the hypothesis that there is a significant degree of underdiagnosis among this subset of the population. It is also hoped that this research will pave the way for more robust studies that can quantify the rate of underdiagnosis and clarify the reasons for it.

## Methods

We carried out a cross-sectional study which included all consecutive female patients who attended one of the International Health Units of the North Metropolitan Barcelona Health Region for pre-travel counseling between June 2016 and June 2021. The inclusion criteria entailed showing signs and symptoms of *Schistosoma* infection and/or having a background that suggested likely past exposure to the *Schistosoma* parasite, including having been born in an endemic country. Patients with previous diagnosis or treatment for *Schistosoma* infection were excluded. For each patient included in the study, her clinical history (including laboratory and ultrasound explorations), records of all previous contacts with the health system's Sexual and Reproductive Health (SRH) units were obtained from the electronic data kept by the health services provider. Additional basic sociodemographic information including country of origin and years of residence in the EU was obtained by means of a questionnaire, which included a checklist of the most commonly recognized signs and symptoms of *Schistosoma* infection and FGS (8–11). Exposure to schistosomiasis was confirmed using the standard ELISA SCHISTO-96 serological test (SciMedx Corporation, Denville, NJ, USA) on a blood serum extracted from participants. Cross-check examination of urine and stool samples was not performed since it did not add substantial benefit to the screening strategy due to the very low yield (3, 4). Proportions were calculated considering as a denominator participants with corresponding data available and stratified by serology results, as shown in Table 1. To detect significant differences between groups, we used the Chi-square or Fisher's exact test for categorical variables and the Student *t*-test or Mann–Whitney *U*-test for continuous variables, where appropriate. Logistic regression was used for multivariate analysis when necessary. Statistical significance was set at *p* ≤ 0.05. Statistical Package Stata 14.0 was used for all statistical analyses. The study was approved by the Ethics Committee of the Germans Trias i Pujol University Hospital, the referral hospital for the study area. Consent was obtained from all study participants. All women with *Schistosoma* diagnosis received appropriate treatment with praziquantel, as well as treatment for other concomitant conditions (i.e., sexually transmitted infections, STIs).

**Table 1:** Baseline sociodemographic information, symptoms of schistosomiasis, gynecological symptoms, laboratory findings and imaging results for 51 sub-Saharan females^a^.

**Variable**	***N* ^b^**	**Overall**	**Serology + (*N* = 30)**	**Serology – (*N* = 21)**	***p*-value**
**Socio-demographic data**					
Age (median, IQR)	51	41.0 [35–48]	40.5 [35–48]	41 [36–48]	0.9
Years in the EU (median, IQR)	51	13 (10-16)	13 (11-18)	13 (9-15)	0.4
Country of origin	51				
Senegal		11 (22.9)	5 (45.4)	6 (54.5)	NA
Mali		9 (18.5)	5 (55.5)	4 (44.5)	
Guinea		5 (10.4)	2 (40.0)	3 (60.0)	
Gambia		13 (27.1)	10 (76.9)	3 (23.1)	
Nigeria		3 (6.25)	3 (100)	0 (0)	
Other		10 (20.8)	6 (60.0)	4 (40.0)	
**Laboratory findings**					
Eosinophilia^c^	50	21 (42.0)	18 (62.1)	3 (14.3)	0.001
Anemia	50	32 (64.0)	24 (82.8)	8 (38.0)	0.001
Hypertransaminasemia of unknown origin	49	9 (18.4)	7 (24.1)	2 (10)	0.3
**Clinical symptoms and findings**					
Chronic abdominal pain	50	31 (62)	27 (93.1)	4 (19.1)	<0.001
Recurring urinary tract infections	49	25 (51.0)	19 (67.9)	6 (28.6)	0.006
Dysuria	48	15 (31.3)	14 (51.9)	1 (4.8)	<0.001
Haematuria	47	11 (23.4)	10 (38.5)	1 (4.8)	0.01
*Gynecological symptoms (overall)*	50	41 (83.7)	27 (96.4)	14 (66.7)	0.02
Vaginal discharge	48	31 (64.6)	22 (81.5)	9 (42.9)	0.006
Vaginal pain	40	11 (27.5)	11 (55.0)	0 (0.0)	<0.001
Vulvovaginal infections	48	31 (64.6)	23 (85.2)	8 (31.8)	0.001
Pelvic discomfort	44	19 (43.2)	18 (78.3)	1 (4.8)	<0.001
Recurrent*Candida albican*s infections	48	30 (62.5)	22 (81.5)	8 (31.8)	0.002
Genital itching	48	29 (60.4)	20 (74.1)	9 (42.9)	0.03
Menstrual pain/dysmenorrhea	44	5 (11.4)	5 (21.7)	0 (0.0)	0.05
Bleeding after sexual inter course	46	14 (21.7)	14 (53.9)	0 (0.0)	<0.001
Amenorrhea	44	9 (20.5)	9 (39.1)	0 (0.0)	0.002
Hypermenorrhea	46	10 (21.7)	7 (28)	3 (14.3)	0.3
Spontaneous abortion	46	11 (23.9)	7 (28)	4 (19.1)	0.5
Infertility	47	11 (23.4)	8 (30.8)	3 (14.3)	0.3
Sandy patches in mucosa	48	2 (4.2)	2 (7.4)	0 (0.0)	0.5
Polyps	48	3 (6.3)	3 (11.1)	0 (0.0)	0.3
Cervical dysplasia	45	4 (8.5)	2 (7.69)	2 (9.5)	0.9
Inflammatory pelvic disease	47	3 (6.4)	3 (11.5)	0 (0.0)	0.2
**Imaging results**					
Abnormal ultrasound transvaginal exploration (overall)	45	15 (33.3)	12 (46.2)	3 (15.8)	0.05
Abnormal ultrasound finding (urogenital and/or hepatosplenic)	33	9 (27.3)	8 (34.8)	1 (10)	0.2
Uterine myoma	45	8 (17.1)	5 (20.8)	3 (14.3)	0.7
Ovarian cysts	44	9 (20.5)	9 (39.1)	0 (0.0)	0.002
Polycystic ovarian	44	6 (13.6)	6 (25)	0 (0.0)	0.03
**Other findings**					
Female genital mutilation	45	9 (20)	6 (23.1)	3 (15.8)	0.7
Asthma	42	5 (11.9)	5 (23.8)	0 (0.0)	0.05
**Coinfections**					
HBV	22	4 (18.2)	2 (16.6)	2 (20.0)	0.9
HPV	30	3 (10.0)	1 (5.0)	2 (20.0)	0.3
HIV	51	1 (2.0)	1 (3.2)	0 (0.0)	0.9
*Strongyloides stercolaris*	44	7 (15.9)	5 (20.8)	2 (10.0)	0.4

a *Global prevalence of study variables are expressed in rows taking as denominator the total number of individuals with this data available, as well as the prevalence of country origin by serology result. All other results consider as denominator the respective number of seropositive and seronegative individuals (columns), excluding those with no data available. Of note, proportions of participants with missing data were similar between seropositive and seronegative women, excluding a recording bias*.

b *N indicates the number of participants with data available*.

c *We excluded a confounding effect of Strongyloides infection and eosinofilia through an adjusted analysis, which yielded an OR between eosinophilia and seropositivity of 9.17 (95%CI = 2.0–42.1; p = 0.004)*.

## Results

Between 2016 and 2021 a total of 405 patients originating from schistosomiasis-endemic countries passed through the screening program, of whom 51 (12.6%) were females, a proportion that contrasts with the total proportion of women among sub-Saharan immigrants (28%) (2). Sociodemographic data and laboratory and imaging results for these women are set out in Table 1. The most frequent country of origin was Gambia (*N* = 13, 27.1%). Of the 51 women screened, 30 (58.8%) had a positive serology, which was comparable to the observed prevalence among men (53.9%, *p* = 0.4). The median age was similar between positive and negative groups of women (40.8 vs. 41, *p* = 0.9) and the median number of years in the European Union was 13 for both groups, without significant differences (*p* = 0.4). Compared to the seronegative women, those with a positive test had a higher prevalence of eosinophilia (62.1 vs. 14.3% *p* = 0.001), anemia (82.8 vs. 38.0%, *p* = 0.001), chronic abdominal pain (93.1 vs. 19.1%, *p* < 0.001), recurring urinary tract infections (67.9 vs. 28.6%, *p* = 0.006), dysuria (51.9 vs. 4.8%, *p* < 0.001), haematuria (38.5 vs. 4.8%, *p* = 0.01) and gynecological symptoms, which were reported by 96.4% of the positive women (vs. 66.6%, *p* = 0.005). The most prevalent gynecological symptoms among infected women were vaginal discharge (81.5 vs. 42.9%, *p* = 0.006), vulvovaginal infections (85.2 vs. 31.8%, *p* = 0.001) and vaginal pain (55.0 vs. 0.0%, *p* < 0.001). Transvaginal ultrasounds showed abnormalities in 12 of the infected women but in only three of the seronegative women (46.2 vs. 15.8%, *p* = 0.05). The most frequent finding was ovarian cysts (39.1 vs. 0.0%, *p* = 0.002) and polycystic ovarian disease (25.0 vs. 0.0%, *p* = 0.03). Other findings include the presence of female genital mutilation in 20% of the women studied (23.1 vs. 15.8%, *p* = 0.7) and bronchial asthma in 23.8% of the infected women (vs. 0.0%, *p* = 0.05). As regards severe complications of schistosomiasis, one patient had liver cirrhosis and another severe chronic renal failure. Both women were seropositive. The median number of previous visits to a SRH unit prior to diagnosis was 41 [IQR (18–65)] and 17 [IQR (11–44)] among seropositive and seronegative women, respectively (*p* < 0.001).

## Discussion

We ascertained a much higher^1^ ^2^ ^3^ prevalence of gynecological signs and symptoms among women with *Schistosoma* seropositive results compared to those with negative results. This finding supports the hypothesis that these women may have a chronic infection of *Schistosoma*. Furthermore, our data revealed some important details. Firstly, the strikingly low number of women that underwent *Schistosoma* screening (in both absolute and relative terms) cannot be attributed to a gender imbalance in the migrant population, but is likely due to an accessibility bias on the part of exposed women to our health service and/or the lack of clinical suspicion when they accessed other preventive or screening services. Records showed that all 51 women had made at least one visit to a SRH unit prior to schistosomiasis screening, and most of them had made more than 50 such visits. This suggests a lack of awareness of FGS among health professionals.

The low proportion of women that underwent *Schistosoma* screening in the study center (51 out of 405, 12.6%), is consistent with previous series which report an underrepresentation of women among the non-European migrant patients diagnosed with schistosomiasis (28.5% of 748 individuals diagnosed between 1997 and 2010) (12). Other case series in Europe, mainly in Italy and southern Spain, show even lower rates for schistosomiasis diagnosis among women, ranging from 3 to 20% of all cases diagnosed (13–16). These low rates of diagnosis among women may be due to poor access to screening programs, rather than a lower prevalence of the infection in women as compared to men. Secondly, the high prevalence of gynecological symptoms among *Schistosoma* seropositive women may denote a disruption of their sexual and reproductive health wellbeing, which probably also explains the very high number of visits made to SRH services. Symptoms of *Schistosoma* infection may have been persistently misdiagnosed. This is in addition to the unspecific chronic complications, such as renal insufficiency, that *Schistosoma* infections may cause and other unspecific manifestations (non-FGS) due mainly to *S. mansoni* infection. The long-term residence in Europe of most of the infected women (median >13 years) supports the notion that migrant females may experience a high rate of delayed schistosomiasis diagnosis. Delayed diagnosis and underdiagnosis rates have been estimated to be high among migrant populations in general (17, 18). Therefore, migrant females exposed to schistosomiasis could be fairly regarded as “neglected among the neglected”, that is, a doubly neglected subset of the population in Europe. The underlying causes of this neglect may be primarily the lack of awareness among health professionals in the SRH and primary care health services. The fact that such services are poorly tailored for migrant female populations may play a role as well, as has been shown to be the case for preventive and screening services among migrant populations elsewhere in Europe (17, 18). As a result, our perception is that the prevalence of *Schistosoma* spp. infection among women may be heavily underestimated and underdiagnosed not only in endemic countries, but in non-endemic countries as well (19). The frequent diagnosis of STIs may be due to an overlapping of signs and symptoms with FGS. Therefore, we cannot exclude a certain confounding between the two conditions. In addition, the frequent diagnosis of asthma observed among infected women (*p* = 0.05) may be due in fact to a misdiagnosis of the signs and symptoms coming from pulmonary involvement of *Schistosoma* infection, as described elsewhere (20).

This study has some limitations. Firstly, the serological test does not distinguish between present/active and past/resolved infections and between different *Schistosoma* species (*S. haematobium*, S. *mansoni, S. japonicum* and others), and only denotes an exposure to *Schistosoma*. Furthermore, the test has limited specificity (84%) with a Negative Predictive Value of 96%. However, the inclusion of compatible clinical symptoms and/or laboratory abnormalities would increase the Positive Predictive Value. The safety of treatment is clearly weighted with the risk of including false positives, and treatment is also unspecific with regard to individual *Schistosoma* species. The second limitation is that this is a single-center study. However, as a referral unit for imported diseases, the study center records most of the *Schistosoma* diagnoses in our area. Third, some data was collected retrospectively (i.e., in past visits and through clinical histories), which may hamper to some degree the quality of the data recorded. Furthermore, the small sample size does not allow adjustments for potential confounders except for *Strongyloides* infection and eosinophilia (see footnote of Table 1). Finally, because of the cross-sectional study design, we cannot establish a causative link between the gynecological signs and symptoms and serology results, and therefore the diagnosis of FGS is presumptive.

## Conclusions

In spite of the above mentioned limitations, our findings provide sufficient evidence to justify the implementation of more robust and prospective studies. These studies would help to determine the need for gender-orientated screening programs among migrant populations in non-endemic countries. Such strategies could involve, for instance, implementation of adapted clinical questionnaires that include the set of FGS signs and symptoms, specific training for professionals devoted to SRH so that they systematically screen at-risk females for *Schistosoma* infection, a reshaping of services to make them more culturally-sensitive and gender-orientated in accordance with WHO evidence-based recommendations (21), and even community-based interventions (awareness campaigns through community-based associations, outreach programs, etc.). These measures could help prevent serious long-term complications and substantial morbidity due to schistosomiasis, thus making intervention more cost-effective and lowering the burden of disease for the most vulnerable populations.

## Data Availability Statement

The raw data supporting the conclusions of this article will be made available by the authors, without undue reservation.

## Ethics Statement

The study was approved by the Ethics Committee of the Germans Trias Hospital, the reference hospital for the study area.

## Author Contributions

SR and OP-Q were responsible for collecting the data, investigation, writing the first draft, and editing the manuscript. XV was responsible for formal analysis and editing the manuscript. IL-M, AT, and AC were responsible for collecting data and investigation. LS and LV were responsible for data curation and investigation. GF-R was responsible for the microbiological sample procedure. BC was responsible for project administration. All authors contributed to the article and approved the submitted version.

## Conflict of Interest

The authors declare that the research was conducted in the absence of any commercial or financial relationships that could be construed as a potential conflict of interest.

## Publisher's Note

All claims expressed in this article are solely those of the authors and do not necessarily represent those of their affiliated organizations, or those of the publisher, the editors and the reviewers. Any product that may be evaluated in this article, or claim that may be made by its manufacturer, is not guaranteed or endorsed by the publisher.

## Footnotes

^1^ https://www.who.int/data/gho/data/themes/topics/schistosomiasis

^2^https://www.who.int/news/item/20-07-2018-female-genital-schistosomiasis-simu ltaneous-screening-of-diseases-can-improve-reproductive-health (accessed October 2021).

^3^https://www.who.int/health-topics/schistosomiasis#tab=tab_3 (accessed October 2021).
